# Fracture Resistance Comparative Analysis of Milled-Derived vs. 3D-Printed CAD/CAM Materials for Single-Unit Restorations

**DOI:** 10.3390/polym15183773

**Published:** 2023-09-15

**Authors:** Cristian Abad-Coronel, Manuel Bravo, Salomé Tello, Emilia Cornejo, Yirelly Paredes, Cesar A. Paltan, Jorge I. Fajardo

**Affiliations:** 1 CAD/CAM Materials and Digital Dentistry Research Group, Faculty of Dentistry, Universidad de Cuenca, Cuenca 010105, Ecuador; estuardo.bravo@ucuenca.edu.ec (M.B.); yirellyparedes@gmail.com (Y.P.); 2 Faculty of Dentistry, University of Cuenca, Cuenca 010105, Ecuador; salome.tello@ucuenca.edu.ec (S.T.); ana.cornejoa@ucuenca.edu.ec (E.C.); 3 New Materials and Transformation Processes Research Group GiMaT, Universidad Politécnica Salesiana, Cuenca 010105, Ecuador; cpaltan@ups.edu.ec (C.A.P.); jfajardo@ups.edu.ec (J.I.F.)

**Keywords:** fracture resistance, CAD/CAM materials, PMMA, 3D-printing, interim restorations

## Abstract

The aim of this study was to evaluate and compare the fracture resistance of a single-unit fixed prosthesis, using a CAD/CAM PMMA material and two printed materials (3DPPa and 3DPPb). A typodont with a specific preparation for a full crown was used; a digital impression was made with a state-of-the-art scanner (PrimeScan^TM^, Dentsply-Sirona^TM^, New York, NY, USA), and a full coverage restoration was designed using a biogeneric design proposal by means of specific software (InLAB 22.1, Dentsply-Sirona, NY, USA). Sixty crowns were prepared, divided into three groups according to the material: 3DPPa (*n* = 20), 3DPPb (*n* = 20), both 3D-printed from the .STL file with a resolution of 50 μm, and PMMA (*n* = 20) milled-derived, which were subjected to a thermocycling process. A universal testing machine (Universal/Tensile Testing Machine, Autograph AGS-X Series) with integrated software (TRAPEZIUM LITE X) equipped with a 20 kN load cell was used to determine the fracture resistance. Significant differences were found by Kruskal–Wallis test and multiple comparisons (*p* < 0.05) in fracture resistance between materials. The fracture resistance for the PMMA material was higher, and the standard deviation was lower (x = 1427.9; sd = 36.9 N) compared to the 3DPPa (x = 1231; sd = 380.1 N) and 3DPPb (x = 1029.9; sd = 166.46 N) prints. The restorations from the milled-derived group showed higher average fracture resistance than the provisional restorations obtained from the printed groups. However, the results demonstrated that all three materials analyzed in single-unit restorations are capable of withstanding the average masticatory forces.

## 1. Introduction

New technologies in dentistry have enabled digital scanning of the tooth preparation within the patient’s oral cavity, the design and fabrication of a custom-made restoration and its placement in a reduced time [1]. This digital process offers greater patient convenience and less margin for error compared to conventional prosthodontic methods that can be more complex and time-consuming [2]. A broad and diverse technology, 3D-printing includes digital light processing (DLP), which uses a light source to create objects layer-by-layer [1]. In addition to DLP, there are other types of 3D-printing, such as stereolithography (SLA), material injection (MI), material extrusion (ME), binder injection, selective laser melting (SLM), sheet lamination and direct energy deposition. All of these methods are available for printing objects and are used in various applications in industry and scientific research [3].

Currently, subtractive milling is a widely used method in computer-aided manufacturing in dentistry and has been shown to be suitable for the production of intraoral prostheses [2]. On the other hand, additive manufacturing is experiencing exponential growth and is likely to be increasingly used in dentistry as its accuracy and variety of applications develop. However, it should be considered that both techniques may present defects during the fabrication process [3,4].

As an additive technique, 3D-printing has several advantages over milling as a subtractive technique. The main advantage is that it allows the creation of more complex or geometrically enhanced 3D-shapes, as it is not limited to shapes that can be cut from a solid block of material. On the other hand, 3D-printing builds the object layer-by-layer, which allows the creation of more intricate shapes with finer details [3,5]. In addition, the additive technique produces less waste than the subtractive technique, using a necessary amount of material to create the restoration. This makes 3D-printing a more sustainable and ecologically friendly option [3].

A number of new materials can be processed by computer-aided design and computer-aided manufacturing (CAD/CAM) technology, used in dentistry for the manufacture of dental prostheses, such as crowns, bridges and veneers, among others, and are characterized by their high precision and ability to produce dental restorations with adequate optical, biocompatible and esthetic properties [6].

Some of the most commonly used CAD/CAM materials in dentistry are ceramic-based and polymer-based materials [7]. All the materials analyzed in this study can be processed by CAD/CAM technology, digitally designed and fabricated with the use of a milling machine or a 3D-printer and are described more specifically below.

### 1.1. PMMA

It is a polymer (synthetic material) obtained by additive or subtractive technique. It is used in several areas such as dentistry, medicine and engineering. In dentistry, it is useful for the elaboration of total and partial prostheses, artificial teeth, among others [8]. Numerous studies have shown that PMMA has significant improvements in mechanical properties, including hardness and fracture resistance [2,9,10,11,12]. As a disadvantage, they have inferior mechanical properties compared to other 3D-printing materials. For this reason, their use would be limited to temporary restorations and not to definitive full-coverage restorations [13]. PMMA is biocompatible and nontoxic; however, by monomers’ elution, possible allergic reactions or inflammation in the patient’s oral tissue should be considered [2,9]. It is less resistant to fracture than other materials, can be easily adjusted and polished, but is prone to plaque build-up and staining. It is esthetically pleasing and can be stained to match the shade of natural teeth [11]. In addition, it is less expensive than some other materials used in the fabrication of dental restorations [14].

### 1.2. 3DPPa

According to its manufacturer, it is a hybrid material, used in the 3D-printing of permanent single crowns, inlays, onlays and veneers; it would be characterized by its ability to offer a precise fit and reproducible results, thanks to its manufacturing process. It is a strong, durable and esthetic material that would allow clinicians and dental technicians to fabricate customized restorations with precision and less time [15]. Additional features of this material include its classification as a Class II medical device, excellent marginal integrity, ease of handling and polishability. It is also formulated to be radiographically visible, which facilitates follow-up dental treatment. The material can be fixed with standard self-adhesive cement and is noted for its high longevity, wear resistance and low porosity. It is suitable for the fabrication of single crowns and is available in seven different shades for optimal esthetic results [14,15]. It is important to consider its mechanical strength and biological compatibility, in addition, precision in the impression process and fit, as well as the durability of the material in relation to occlusal forces and abrasion resistance [14]. The technology of 3D printing can reduce the cost of the material used in the fabrication of customized dental restorations and speed up post-production processes compared to other methods. In addition, the speed of the impression could be less than 20 min in some cases [15].

### 1.3. 3DPPb

According to the manufacturer, it is a 3D-printable polymer that features an optimal combination of translucency and opacity to mimic natural dentition. The ceramic content would provide superior esthetics, reducing the number of visits and chair time. It features a clinically advanced formula that requires minimal resin preparation and up to 7 times faster finishing. It is the first 3D-printable polymer for restorative dentistry that is fully radiopaque, has high condensed ceramic content and is easy to characterize [16].

Therefore, considering the recent advances in technology, in the introduction and use of different materials in the fabrication of fixed dental prostheses, the present study aimed to evaluate and compare the fracture resistance of single full-coverage restorations in three different materials: two of them 3D-printed and one in PMMA, all materialized by means of a CAD/CAM system.

## 2. Materials and Methods

### 2.1. Sample Preparation

Sixty crowns were used, divided into three groups of 20 each, according to the material used. The materials used in the study are shown in Table 1.

**Figure 1:**
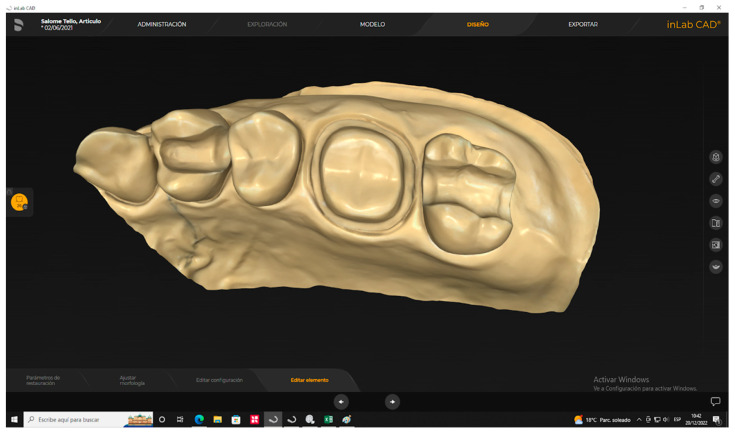
Maxillary typodont with preparation for a crown in Section 2.6.

The protocol used for crown preparation on tooth 2.6 consisted of occlusal reduction of 2 mm and an axial reduction of 1 mm. In addition, a chamfer line was established to ensure a smooth transition between the crown and the natural tooth. In order to maintain a consistent shape, the axial walls of the tooth were prepared with a parallelism of 6 to 10 degrees, and the edges were rounded (Figure 1).

### 2.2. PMMA Samples

Crowns with the PMMA material were obtained using the CNC (computer numerical control), a machine used in the industry for the milling process. The CNC milling process allowed the fabrication of crowns from a 3D-digital model of the CAM software (InLab CAM, 20, Dentsply-Sirona, New York, NY, USA). Once the dental prosthesis was designed in software, the file was sent to the integrated milling unit (MCX5, Dentsply-Sirona, New York, NY, USA) to produce the prostheses.

### 2.3. 3D-Printed Crowns

#### 2.3.1. Digitalization Scanning and Design of Samples

**Figure 2:**
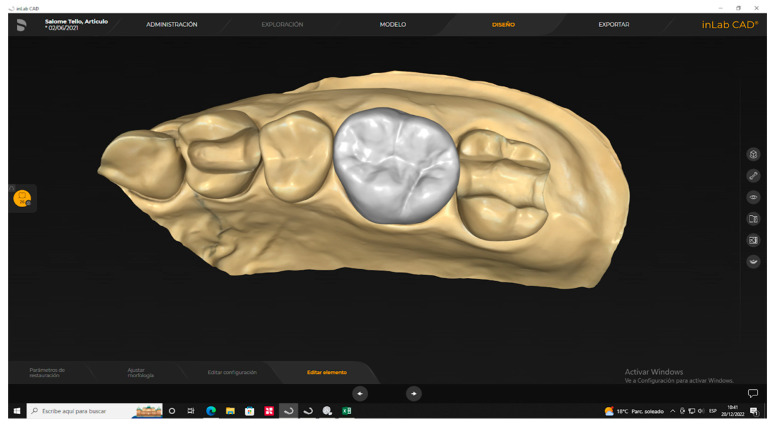
Design of a fixed dental crown using InLAB 20.0, Dentsply-Sirona’s biogeneric mode.

A scanner (PrimeScan 2.0, Dentsply-Sirona, New York, NY, USA) was used to obtain a digital impression of the prefabricated model. Subsequently, the digital model was processed using a complex design software (InLAB 20.0, Dentsply-Sirona, New York, NY, USA) to create an indirect restoration using the biogeneric mode (Figure 2).

#### 2.3.2. 3D-Printed Materialization

The samples were printed at a resolution of 50 μm by transferring the same CAD design in an STL (Standard Triangle Language) format file to the CAM software of the 3D-printer (Pro-95, SprintRay, Los Angeles, CA, USA).

#### 2.3.3. Postproduction

The printed samples were immersed in ninety percent (90%) alcohol to remove resin residues for ten (10) min in a printer integrated apparatus created for this purpose (SprintRay Pro Wash/Dry, SprintRay, Los Angeles, CA, USA). Subsequently, they were subjected to a light curing process under UV (ultraviolet) light for nine (9) min in an automatically integrated source (SprintRay Pro Cure, SprintRay, Los Angeles, CA, USA).

### 2.4. Thermocycling of Samples

The milled-derived and printed samples underwent a thermocycling process consisting of 5000 cycles. This was carried out using a computerized thermal cycler (Thermocycler™, SD Mechatronik, Feldkirchen-Westerham, Germany) that kept the samples in extreme thermal cycles of 5 °C and 55 °C in distilled water, each cycle lasting 25 s and a pause time of 10 s. After each loading phase, the samples were dried and thoroughly inspected for cracks, chips or fractures, in order to ensure the strength and quality of the samples.

### 2.5. Fracture Resistance Test

**Figure 3:**
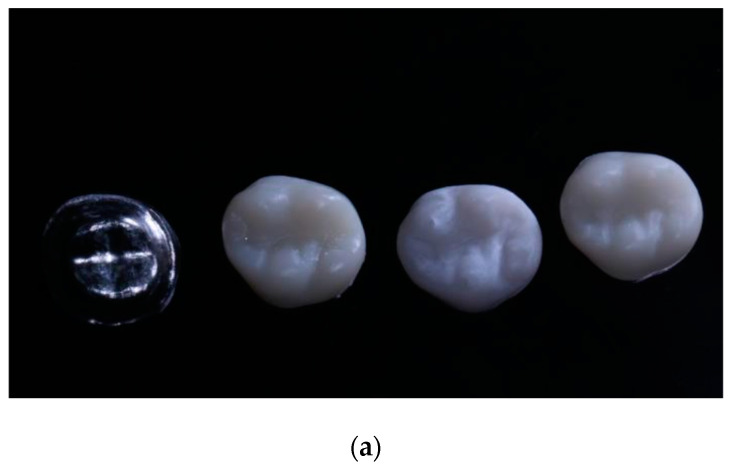

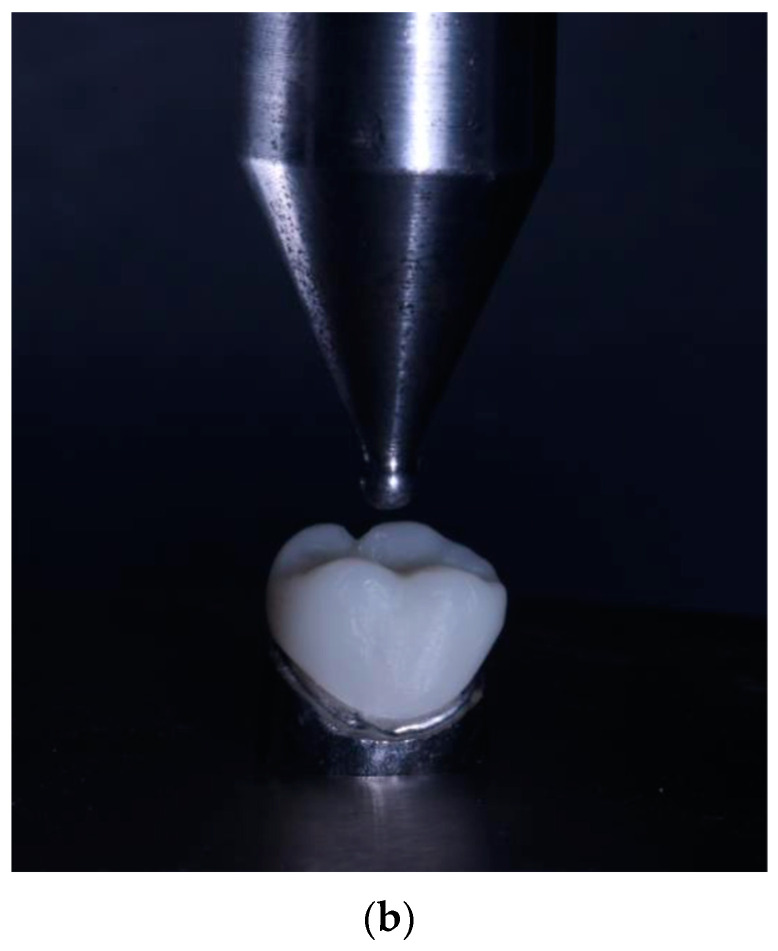
(**a**) (From left to right) Metal stump, PMMA, 3DPPb and 3DPPa crowns. (**b**) Fracture resistance test.

**Figure 4:**
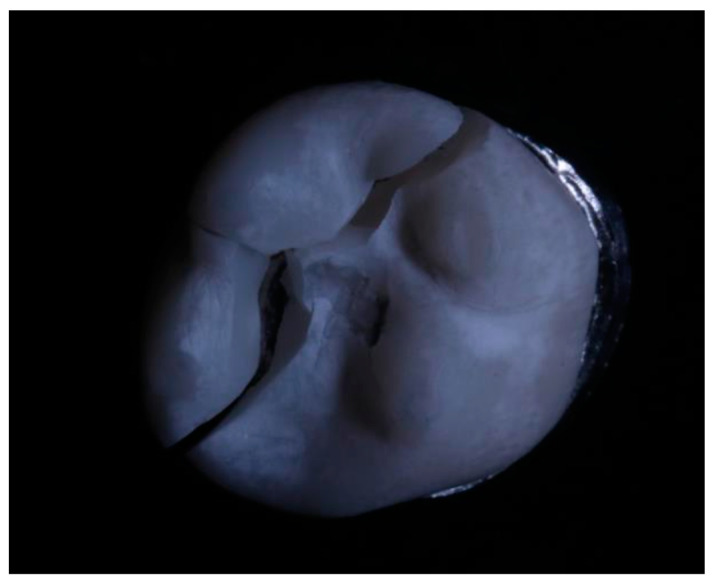
Fractured 3DPPb crown.

The fracture resistance of the crown was evaluated by means of an experimental design carried out under laboratory conditions. Each printed crown was adapted to a metal die that was attached to the platform of a universal testing machine (Shimadzu AGS-X series Universal Testing Machine; Shimadzu, Tokyo, Japan), equipped with a 20 kN load cell. A load was applied to each specimen at a rate of 0.5 mm/min in the direction parallel to the major axis of the tooth, with an initial preload of 10 N. To carry out the test, a hardened steel pilot punch was used and applied to the central pit of the restoration until fracture occurred. The applied loads were recorded in Newtons (N) with a sensitivity of 0.1% (Figure 3a,b and Figure 4).

### 2.6. Data Processing and Analysis

A statistical program (SPSS v.25, IBM, New York, NY, USA) was used for data analysis. A descriptive and graphic summary was made of the measurements obtained for each material; normality and homoscedasticity tests were performed; the Kruskal–Wallis statistic and posteriori comparisons were used for hypothesis testing. A significance level of 5% was used.

## 3. Results

### Descriptive Analysis

The highest average fracture resistance value was reported for the PMMA material (x = 1427.9 N; sd = 36.9 N), whilst the variability between repetitions was low (cv = 3.2%) with a minimum fracture toughness of 1368.8 N and a maximum of 1486.8 N. Followed by the 3DPPa material (x = 1231.0 N; sd = 380.1 N), the variability between replicates was high (cv = 30.9%), and the minimum and maximum values reported were 622.8 N and 1848.4 N, respectively. The 3DPPb material reported the lowest mean value (x = 1029.92 N; sd = 166.4 N), the variability between replicates was medium (cv = 16.2%), the minimum resistance reported was 786.1 N and the maximum 1282.2 N. The results of this mechanical experiment are shown in Table 2.

No outliers were reported in the fracture resistance measurements of the three materials. In terms of quartiles for the 3DPPa material, 25% of the measurements were less than 887.5 N, 50% less than 1308.1 N and 75% less than 1517.1 N. For the 3DPPb material, 25% of the fracture resistance values were less than 857.4 N, 50% less than 1037.2 and 75% less than 1180.9 N. For the PMMA material, 25% of the values were less than 1391.9 k, 50% less than 1429.9 N and 75% less than 1456.7 N. Higher variability was found in the measurements of the 3DPPa material, medium variability in the measurements of the 3DPPb material and lower variability in the measurements of the PMMA material.

**Figure 5:**
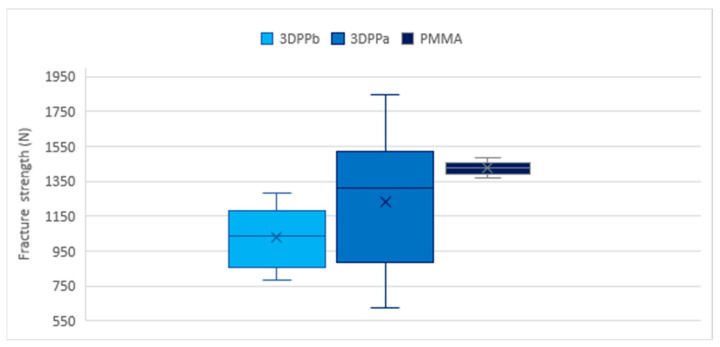
Box plot for fracture resistance of 3DPPa, 3DPPb and PMMA. Quartile graph, maximum and minimum values, mean (x) and median (central line).

From multiple comparisons, it was determined that the mean fracture resistance obtained with the PMMA material differs from the mean resistance obtained with the 3DPPb (*p*-value < 0.05) and 3DPPa (*p*-value < 0.05) materials. Statistically significant differences were also found in the mean fracture resistance between the 3DPPb and 3DPPa materials (*p*-value < 0.05). Results are shown on Table 3 and Figure 5.

The fracture resistance values met the assumption of normality (*p*-value > 0.05) but did not meet the assumption of homoscedasticity (*p*-value < 0.05); therefore, for the comparison of mean fracture resistance between materials, the Kruskal–Wallis nonparametric test was used. According to the test result, the null hypothesis that the fracture resistance distribution is the same between material type categories was rejected (h = 23.061; *p*-value < 0.05).

## 4. Discussion

The present investigation aimed to analyze the fracture resistance of full-coverage unitary fixed prostheses using subtractive and additive techniques. The null hypothesis was posed that there would be no significant difference in fracture resistance between 3D-printed and milled-derived fixed prostheses. An analysis of the fracture resistance of PMMA (milled), 3DPPa and 3DPPb (3D-printed) materials was performed using a computer-aided design and manufacturing system. The results allowed the null hypothesis to be rejected, indicating that there are significant differences in fracture resistance between the different materials evaluated.

Currently, subtractive fabrication methods generate more uniform objects, making them a more appropriate choice for the production of intraoral prostheses that need to withstand higher occlusal loads [4]. On the other hand, additive manufacturing methods offer the possibility of creating larger objects with surface irregularities, cavities and hollow shapes, making them ideal for the creation of facial prostheses and metal frameworks in removable partial dentures [12]. It is undeniable that computer-aided manufacturing procedures will transform various aspects of dentistry in the future, especially in terms of simplicity of treatment and speed of production [4,12,17].

When comparing the fracture resistance in our study, the PMMA material (milled-derived) obtained the highest average value of fracture resistance and lowest scatter (m = 1427.94 N; sd= 36.93 N), followed by the 3DPPa printed material (x = 1231.0 N; sd= 380.1 N) and the 3DPPb printed material (x = 1029.9 N; sd = 166.46 N). According to the coefficient of variation, the PMMA material showed higher accuracy, followed by the 3DPPb material, and the 3DPPa material showed lower accuracy between measurements. The results show that, for fixed prosthetic restorations, the milled-derived crowns had higher strength than the crowns obtained by 3D-printing. However, the variations were minor, highlighting their consistency in the values obtained for printed materials.

The results are in agreement with those obtained in a study by Martin Ortega et al. [18] where implant-supported anterior temporary crowns can be fabricated by subtractive or additive manufacturing procedures; however, milling procedures produced stronger implant-supported temporary crowns than the additive manufacturing methods tested. Also, in another investigation [19], the authors concluded that CAD/CAM milling and 3D-printing of temporary restorations may represent favorable options for long-term provisionalization, because temporary crowns created using the CAD/CAM method and traditionally fabricated dimethacrylates demonstrated significantly higher fracture resistance compared to conventionally fabricated monomethacrylate resins after the aging process.

In another study [20], higher values were reported for milled-derived crowns compared to 3DPPa crowns. Other authors [21] evaluated the fracture resistance of fixed dental prostheses, high-density polymers, fiber-reinforced composite and metal-ceramic, using the subtractive method for the fiber-reinforced composite and high-density polymers groups, and the additive method for the others. The metal-ceramic group reported the highest fracture resistance with a statistically significant difference compared to the other groups. No significance was observed between the 3DP and high-density polymers groups while the fiber-reinforced composite group showed the lowest value. The highest frequency of non-repairable failures was observed in the metal-ceramic and fiber-reinforced composite groups, while the high-density polymers and 3DP groups reported a high frequency of repairable failures.

Studies that compared the physical properties of PMMA CAD/CAM with the conventional group polymerized by heat showed that it had significant superiority in surface wettability, surface roughness and hardness [9,12,22]. The homogeneous heating of PMMA results in higher monomer conversion, reduces the plasticizing effect of residual monomers and consequently increases surface hardness [23]. In comparison, 3D-printed temporary crowns have superior mechanical properties, but inferior physical properties compared to CAD/CAM milling and other conventionally fabricated ones [24,25,26]. Temporary crowns made from 3D-printed can be used as an alternative to conventional and CAD/CAM milled-derived long-lasting temporary materials [27,28].

However, the results found in the present investigation differ from another study [29] where, when evaluating the fracture resistance and failure pattern of milled-derived and 3D-printed composite resin crowns as a function of different material thicknesses, the crowns of the 3D-group showed the highest values of fracture resistance compared to the milled-derived group within the three thicknesses tested. It should be noted that in addition to the different thicknesses studied, the specimens were adhesively cemented to resin cores. Thus, this polymer–die–resin bonded complex could have generated high strength values, which differs from our method, in which we tried to specify the fracture resistance value exclusively relative to the material.

Jockin et al. compared several materials with natural dentin (since most of the tooth is dentin) including a 3DPPa resin and did not observe fractures in the material in any of the cases, which ratifies that the printable polymer is a viable material for single crowns [30]. This result is favorable for 3D-printable polymer as they offer mechanical performance comparable to that of natural dentin, as they exhibit similar physical and mechanical properties. These properties include compressive strength, flexibility and the ability to withstand chewing loads. Although, fixed prosthetic restorations and milled-derived crowns have higher strength than crowns obtained by 3D-printing.

In another investigation [31] on crowns with the 3DPPa material, the authors demonstrated that before the 10-year chewing simulation, the average breaking load value of 3DPPa crowns was 1936 N, a value that was unchanged after the chewing simulation. Tests show that crowns made with 3DPPa show breaking loads twice as high as the maximum average human masticatory forces of 720 N, both initially and after a 10-year mastication simulation [20]. The average breaking load value for the 3DPPa crowns of 1936 N obtained by other authors [20] has been found to be higher than the one found with the present study of 1231.1 N, where the milled-derived and printed samples underwent a thermocycling process consisting of 5000 cycles, in extreme thermal cycles of 5 °C and 55 °C.

In another study [32], the effect of thermocycling on the fracture resistance of temporary restorations created by computer-assisted 3D-printing and milling methods was evaluated. The authors found that temporary crowns fabricated with 3D-printing showed higher fracture resistance compared to temporarily milled-derived crowns. Considering the effect of thermocycling, this study differs from the findings found by the present investigation, because the mean fracture resistance obtained with 3DPPa and 3DPPb printed crowns resulted lower compared to the mean fracture resistance obtained with milled-derived crowns.

According to other authors [33], three-dimensional additive manufacturing (AM) technology is undergoing constant development in the field of dentistry, presenting itself as an alternative to conventional subtractive milling (MM) and traditional processing methods. AM shows comparable characteristics to MM in terms of mechanical properties, especially in polymeric materials. However, it has been observed that the fracture resistance of prostheses printed by AM is still lower than that obtained by conventional and MM techniques. Consequently, prostheses fabricated by AM are most often used for temporary crowns and fixed partial dentures, because their stiffness and fracture resistance are not adequate to withstand chewing forces for prolonged periods of time. However, for single crowns, the values shown may suggest that the printed materials can be integrated into a stable collusion with predictable results.

It is important to note that the present investigation showed several limitations. One of the main ones was that the experiment was conducted under in vitro conditions rather than in a clinical oral setting. This difference clearly influences the results and, therefore, their interpretation should be subject to more rigorous scrutiny. This is because testing methods in the field of dental biomaterials are still predominantly traditional, based on real experiments, which presents a challenge due to the time, costs, and resources required to perform extensive in vitro test series [34,35,36]. It should also be noted that the tests have been performed thanks to the advances in digital dentistry where the samples can be exactly standardized, simulating the anatomy and morphology of the restorations to be evaluated with the clinical thicknesses to be used in the respective material. However, further investigations under highly similar clinical conditions are necessary in order to evaluate the bond strength of CAD/CAM-milled-derived and 3D-printable polymer. These studies should include the exploration of various surface treatments to determine which of them is the most suitable in terms of repairs.

The present research provides evidence that 3D-printed dental crowns using the 3DPPa material can offer adequate long-term strength. These findings support the feasibility of 3D-printing as an alternative to traditional milling techniques for the fabrication of dental crowns [37]. However, it should be considered that the choice of material for dental crowns will depend on the individual needs and requirements of each patient. In addition, 3DPPa and 3DPPb materials are relatively new on the market, which has resulted in a paucity of scientific literature addressing in detail all their properties [38]. Therefore, further studies and time are required to comprehensively analyze these materials. Consequently, subsequent research focusing on in-depth analysis of fracture resistance properties is needed.

## 5. Conclusions

Crowns milled-derived with the PMMA subtractive technique reported higher average fracture resistance and accuracy compared to crowns with the additive 3DPPa and 3DPPb impression technique. However, the latter were within the acceptable range of chewing loads for full-coverage single-tooth restorations. Therefore, the fabrication of printed restorations could be considered as a reliable technique for the fabrication of single-unit final crowns.

## Figures and Tables

**Table 1:** Material used in this study.

Product Name	Manufacturer’s Brand	Lot	Material
3DPPa	SprintRay (Los Angeles, CA, USA)	600,663	Hybrid material for dental 3D-crown printing
PMMA	Ivoclar Vivadent (Lichtenstein)	48,820	Thermoplastic polymer
3DPPb	SprintRay	0202021	Nano ceramic hybrid, 3D-printing

**Table 2:** Descriptive summary of fracture resistance of 3DPPa, 3DPPb and PMMA.

Material	*n*	Mean (x)	Standard Deviation (sd)	Coefficient of Variation (cv)	Minimum	Maximum	Ci 95% for the Mean
PMMA	20	1427.9	36.9	3.2%	1368.8	1486.8	(1410.6; 1445.2)
3DPPa	20	1231.0	380.1	30.9%	622.8	1848.4	(1053.1; 1408.9)
3DPPb	20	1029.9	166.4	16.2%	786.1	1282.2	(952.0; 1107.8)

Note: unit of measurement Newton (N), Material 3DPP a: Crown; 3DPP b: OnX.

**Table 3:** Results of the simultaneous test for differences in means.

Sample 1—Sample 2	Test Statistic	Deviation from Test Statistic	*p*-Value
3DPPb—3DPPa	14.150	2.562	0.010
3DPPb—PMMA	−26.500	−4.798	0.000
3DPPa—PMMA	−12.350	−2.236	0.025

Note: Mann–Whitney U statistic, independent samples, level significance 5%.

## Data Availability

https://drive.google.com/drive/folders/18Lbfc0z9Mxb2qDc81KCkUkEGmzNQP24L?usp=drive_link.
